# Effects of house-cultivated edible bird’s nest on immunoglobulin and cytokine release *in vitro*

**DOI:** 10.14202/vetworld.2024.1370-1384

**Published:** 2024-06-28

**Authors:** Mel June Choong, Hemaniswarri Dewi Dewadas, Lay Cheng Lim, Sheela Devi Sukuru, Chee Hong Tan, Soon Keng Cheong, Yang Mooi Lim

**Affiliations:** 1 Centre for Cancer Research, M. Kandiah Faculty of Medicine and Health Sciences, Universiti Tunku Abdul Rahman, 43000 Kajang, Selangor, Malaysia; 2 Centre for Biomedical and Nutrition Research, Faculty of Science, Universiti Tunku Abdul Rahman, Jalan Universiti, Kampar, 31900, Perak, Malaysia; 3 Department of Business and Public Administration, Faculty of Business and Finance, Universiti Tunku Abdul Rahman, Jalan Universiti, Kampar, 31900, Perak, Malaysia; 4 Department of Life Sciences, School of Pharmacy, International Medical University, 57000 Kuala Lumpur, Malaysia; 5 Department of Nursing, M. Kandiah Faculty of Medicine and Health Sciences, Universiti Tunku Abdul Rahman, 43000 Kajang, Selangor, Malaysia; 6 Inbit Biotech Sdn. Bhd., No. 8, Lorong University B, Seksyen 16, 46350 Petaling Jaya, Selangor, Malaysia; 7 Department of Medicine, M. Kandiah Faculty of Medicine and Health Sciences, Universiti Tunku Abdul Rahman, 43000 Kajang, Selangor, Malaysia; 8 Department of Pre-clinical Sciences, M. Kandiah Faculty of Medicine and Health Sciences, Universiti Tunku Abdul Rahman, 43000 Kajang, Selangor, Malaysia

**Keywords:** cytokines, edible bird’s nest, immunoglobulins, modular immune *in vitro* construct model, swiftlets

## Abstract

**Background and Aim:**

Edible bird’s nest (EBN) is known as the “Caviar of the East” because of its high nutritional and medicinal values. Nevertheless, its effect on human immunity is yet to be explored. This study examined the effects of EBN’s aqueous extract (EBNE) on human immunity through the modular immune *in vitro* construct (MIMIC) model consisting of peripheral tissue equivalent (PTE) and lymphoid tissue equivalent (LTE) modules.

**Materials and Methods:**

One hundred twenty mL of full blood was obtained from four healthy human volunteers. The human immune system was simulated using an *in vitro* model, called MIMIC. Under EBNE treatment, monocyte transendothelial migration through reversed endothelial layers was observed. Using PTE and LTE modules, monocytes were differentiated into dendritic cells with lipopolysaccharide, then co-cultured with T- and B-cells for cytokine and immunoglobulin (Ig) production. The human cytokine array G2000 and quantitative human Ig isotyping array were used to identify the cytokine profile and Ig isotypes, respectively.

**Results:**

IgE, IgA, and IgG3 levels were significantly raised by EBNE. These cytokines, including brain-derived neurotrophic factor, ciliary neurotrophic factor, glial cell line-derivative neurotrophic factor, insulin-like growth factor 1, and insulin-like growth factor binding protein 4, were generated.

**Conclusion:**

For the first time, this work uses a MIMIC model to illustrate the impact of EBNE on human immune response. This new understanding of EBN’s immunoregulatory effect allows for further exploration of how EBN interacts with the human immune system.

## Introduction

Major diseases, including infection, cancer, autoimmunity, and allergy, significantly disrupt the normal functioning of the immune system. Continued progress in understanding basic immune mechanisms is essential for developing new abilities to treat and prevent diseases that affect millions of people worldwide [1]. Identifying a safe adjuvant medicine from natural products could markedly enhance the quality of life for immunocompromised patients. Experts and scholars around the world have given significant attention to this topic. The immune-boosting capabilities of nutraceuticals, verified by multiple preliminary studies [2–4], are eagerly awaited today. Swiftlets’ nests, referred to as “Yanwo” in Chinese, are edible. Since the past 16 centuries, it has been valued as a precious food tonic. Edible bird’s nest (EBN), famously referred to as the “Caviar of the East,” boasts a premium price and exceptional nutritional value [5]. The sublingual salivary glands of male swiftlets generate saliva used in constructing EBN during breeding season [6]. Swiftlets in the *Apodidae* family belong to the genera *Aerodramus* and *Collocalia* [7]. For thousands of years, the Chinese have utilized this distinctive food tonic as a traditional Chinese medicine (TCM), renowned for its health-boosting properties. EBN exhibits diverse health benefits, including enhancing skin complexion, fortifying the immune system, treating tuberculosis and other diseases, and alleviating various conditions such as asthma, stomach ulcers, gastric troubles, and bronchial ailments [5, 8–12].

Few scientific studies [13–17] have documented the medical and health benefits of consuming EBN, despite its numerous pharmaceutical uses and health claims. The medicinal benefits of EBN are established due to its multiple bioactivities resulting from the presence of amino acids, glycoproteins, and sialic acid. Glycoproteins and sialyl glycoconjugates function in cell proliferation, wound healing, and immune response. Ng *et al*. [13] initially proved that the water extract of EBN could boost human peripheral blood monocytes’ mitogenic response, thereby enhancing immunity. 3T3 fibroblasts’ DNA synthesis could be stimulated by the epidermal growth factor (EGF)-like activity identified in EBN extracts [14]. Zhang *et al*. [15] proved that pearl powder’s EBN enhanced DNA synthesis in T lymphocytes and boosted serum IgM levels in mice. The suggested effect of EBN on immunity could be enhanced. According to Guo *et al*. [6], the EBN extract was effective in binding to influenza viruses, inhibiting their hemagglutination on erythrocytes, and neutralizing influenza virus-infected Madin-Darby canine kidney cells. According to Yew *et al*. [16], their EBN extracts displayed neuroprotective actions against neurodegenerative disorders, including Parkinson’s disease, triggered by oxidative stress. Murugan *et al*. [17] have demonstrated that hydrolyzed EBN inhibits oxidative stress and preserves endothelial function in hyperglycemic conditions, rendering it useful for diabetes-related micro- or macrovascular complications management. In addition, EBN contains 18 of 20 types of amino acids [18] that are needed by humans. Aspartic acid and serine are the primary water-soluble amino acids in EBN, as reported in several studies [5, 18, 19]. Amino acids play critical roles in cellular energy production, regulating cell function, and bolstering the immune system by generating immune globulins and antibodies. Dobutr *et al*. [12] revealed that EBN promotes T-cell activation by influencing major histocompatibility complex class II and costimulatory molecule stimulation during T cell receptor/pMHC-II interaction. A comprehensive understanding of how EBN influences the immune system remains elusive. These claims lack substantial clinical evidence. The high nutritional and therapeutic content of EBN suggests its potential for boosting immunity.

This study investigated the impact of EBN’s aqueous extract (EBNE) on human immunity through monocytes, dendritic cells (DCs), T-cells, and B-cells through the peripheral tissue equivalent (PTE) and lymphoid tissue equivalent (LTE) modules of the modular immune *in vitro* construct (MIMIC) model. This study’s results offer detailed insights into how EBN influences the human immune system. Its medicinal value for human immunity could be substantiated by this.

## Materials and Methods

### Ethical approval and Informed consent

This study was approved by the UTAR Scientific and Ethical Review Committee (UTAR-SERC) with ethical approval number - U/SERC/15/2014. All participants provided written informed consent prior to their enrollment in the study.

### Study period and location

This study was conducted from October 2018 to May 2023 at Centre for Cancer Research, M. Kandiah Faculty of Medicine and Health Sciences, Universiti Tunku Abdul Rahman, 43000 Kajang, Selangor, Malaysia.

### EBN sample

The EBN samples used in this study were produced by the swiftlet *Aerodramus fuciphagus* from the *Apodidae* family. The house-cultivated EBN samples were provided by Inbit Biotech Sdn. Bhd. and was harvested from Pahang state, Malaysia,

### EBNE preparation

Raw EBN was soaked in deionized distilled water and the feathers in the nests were manually removed using forceps. At 37°C, the nests were dried and ground into a fine powder in a mortar. The EBN powder was soaked in deionized distilled water for 24 h. Each extract was boiled for an hour in a water bath. The EBNE was filtered through filter paper after being centrifuged at 1957× *g* for 15 min and cooled. The filtrate was freeze-dried and kept at –20°C for storage.

### Human umbilical vein endothelial cell (HUVEC) cell and culture conditions

HUVECs obtained from the American Type Culture Collection, Virginia, USA, were seeded at 5000 cells/cm^2^ density and maintained at 75 cm^2^ plastic cell culture flasks (SPL Life Sciences, Gyeonggi-do, Korea). Cells were cultured in VascuLife® EnGS Medium (Lifeline® Cell Technology, Frederick County, Maryland, USA) with 10% fetal bovine serum (FBS) (Capricorn Scientific, Ebsdorfergrund, Germany) and 1% antibiotic solution (Gibco, Grand Island, New York, USA). The cells were incubated at 37°C with 5% CO_2_, 95% air, and 95% relative humidity, with media replacement every 2 days.

### WST-8 cell proliferation assay

The cell count reagent WST-8 [2-(2-methoxy-4-nitrophenyl)3-(4-nitrophenyl)-5-(2,4-disulfophenyl)-2H-tetrazolium, monosodium salt] assay (Nacalai Tesque, Kyoto, Japan) was used to evaluate the influence of various EBNE concentrations on DC proliferation. The WST-8 reagent in cell count reagent SF generates water-soluble formazan dye upon reduction, which was used in this assay with DCs collected from the PTE module. 1 × 10^5^ cells were cultured in Roswell Park Memorial Institute (RPMI)-1640 medium (Gibco Life Technologies, Waltham, MA, USA) with 20% FBS in 96-well plates (Nest Biotechnology, NJ, USA). 24 h post-incubation, the medium was switched to one without FBS and added EBNE at concentrations of 20, 40, 60, 80, and 100 μg/mL for 72 h, followed by adding 10 μL of WST-8 solution. The cells were then incubated for 3 h at 37°C. Using a microplate reader, Model Infinite M200 Pro (Tecan, Männedorf, Switzerland), the absorbance of formazan generated from WST-8 was assessed at a wavelength of 450 nm.

### Human autologous serum preparation from whole blood samples

One hundred twenty mL of blood was collected from each donor. Blood samples were gathered from a group of 4 donors (age range: 18–32), all of whom were non-smokers, non-alcoholics, and non-addicts. Donors had no chronic diseases, inflammatory conditions, or major surgeries in the preceding 4 weeks, and had not undergone any immune therapy within the previous year. 120 mL of blood was collected, 60 mL was dispensed into a Terumo syringe (Terumo, Tokyo, Japan) without anticoagulant, 20 mL was transferred to BD tubes with ACD Sol A (Becton Dickinson, New Jersey, USA) for monocytes, and 40 mL went to BD tubes with ACD Sol A (Becton Dickinson) for T- and B-cell isolation. 60 mL blood was carefully transferred into two 50 mL conical tubes (Nest Biotechnology) to allow serum separation for 30 min. A sterile pipette from SPL Life Sciences in Gyeonggi-do, Korea, was used to extract blood, which was then allowed to coagulate for an hour. The tube holding congealed blood was spun at 400× *g* for 15 min. The supernatant was obtained by centrifuging the yellow fraction of the blood at 704× *g* for 15 min. The serum was heated to 59°C for 30 min. Monocytes were incubated in a medium prepared with freshly prepared serum [20–22].

### Media preparation for monocytes and T- and B-cell cultures

Monocyte, T- and B-cell culture media were prepared with RPMI-1640 enriched by 20% heat-inactivated human autologous serum and 1% antibiotic (penicillin-streptomycin) solution. All media were filtered through 0.22 μm membranes (Merck Millipore, Billerica, Massachusetts, USA). The media used for culturing were prepared freshly, while the rest were stored at 4°C for future utilization.

### Human monocytes preparation through StraightFrom™ whole blood CD14 microbeads

Twenty mL of blood was collected under aseptic conditions and transferred into BD vacutainer tubes (ACD Sol A). The tubes were inverted five times to thoroughly mix the blood with anticoagulant before transferring it carefully to a 50-mL conical tube. 1 mL of StraightFrom™ Whole Blood CD14 MicroBeads (Miltenyi Biotec Inc., Auburn, California, USA) was incubated with the blood in an ice-chilled mini-shaker (Biosan Laboratories Inc., Warren, Michigan, USA) at 10 rpm for 30 min. Twenty mL of separating buffer was added to the blood. The blood cells were spun down at 445× *g* for 10 min. At the bottom of the tube, the monocyte-containing layer had a reddish hue. Ten mL of separating buffer was added to top off the yellowish supernatant. Four mL of separating buffer was used to condition the blood column before adding the blood sample. Forty mL of separating buffer was used to rinse the blood column. A 15-mL centrifuge tube was used to hold the blood column. Five mL of elution buffer was pipetted into the blood column and the monocytes were eluted in the dark by pushing the plunger. Monocytes were centrifuged for 7 min at 176× *g* after elution. One mL of RPMI medium with 20% heat-inactivated human autologous serum was added to the pellet and gently mixed. Monocyte count was determined using the trypan blue exclusion technique. Monocytes were placed above HUVECs in a hanging insert to model innate immune response [20–22].

### Human T-cells preparation through StraightFrom™ whole blood CD4 microbeads

Instead of monocytes, T-cells were prepared using StraightFrom™ Whole Blood CD4 MicroBeads following the same procedure. T cells in the whole blood sample were purified using the same procedures described earlier in this article. T-cells were seeded on HUVECs in a hanging insert for an adaptive immune response model, once their cell number was determined [20–22].

### Human B-cells preparation through StraightFrom™ whole blood CD19 microbeads

B-cells were prepared using StraightFrom™ Whole Blood CD19 MicroBeads, following the same procedure used for monocytes. The process for purifying B cells from the whole blood sample was repeated as described earlier. B-cells, after obtaining their cell number, were cultured on HUVECs in a hanging insert to MIMIC an adaptive immune response [20–22].

### Determination of the purity of monocytes, T- and B-cells by flow cytometry analysis

Monocytes were identified using CD14-fluorescein isothiocyanate (FITC) labeled antibodies. CD14-FITC/CD4-PE and CD20-FITC/CD45-PE antibodies were used for T- and B-cell identification, respectively. REAfinity (REA) Control – FITC and REA Control – PE antibodies served as negative controls. The supernatant was aspirated after centrifuging the isolated monocytes, T- or B- cells at 300× *g* for 10 min. Two μL of antibodies were added to the cell pellet suspended in 98 μL of buffer. The cell suspension was incubated in the dark on ice for 10 min. After incubation, the cells were centrifuged at 300× *g* for 10 min to pellet them, and the supernatant was completely aspirated. One mL of buffer was used to suspend the cell pellet before flow cytometry analysis. 98 μL of buffer was added and followed by 2 μL of the respective antibodies. The suspension of cells was incubated in the icebox for 10 min in darkness. The cells were then centrifuged at 300× *g* for 10 min after being washed with 2 mL of buffer and having the supernatant completely aspirated. One mL buffer was used to suspend the cell pellet before flow cytometry analysis.

### *In vitro* PTE module preparation

**Figure-1:**
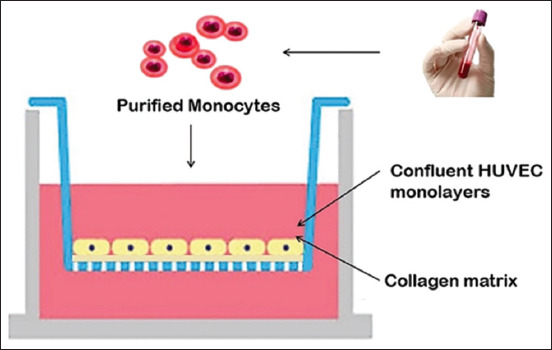
Peripheral tissue equivalent (PTE) module. Innate immune responses were primarily observed in the PTE module. Pre-screened donors were forked over leukocytes and processed into monocytes. The purified monocytes were placed into specially designed “tissue constructs,” which were forged from collagen and endothelial cells. Monocytes then selectively migrated through the endothelium spontaneously and differentiated into subsets of antigen-presenting cells such as dendritic cells or macrophages. Edible bird’s nest (EBN)-treated dendritic cells were reverse transmigrated through the endothelium, mimicking traffic across the lymphatic system. After reverse transmigration, the dendritic cells were exposed to the test EBN extract.

**Figure-2:**
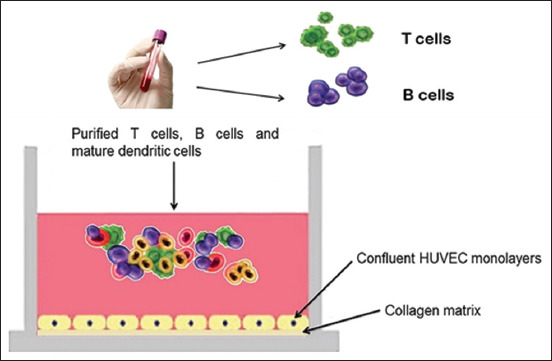
Lymphoid tissue equivalent module in the modular immune *in vitro* construct system. Lymphoid tissue equivalent responses summarize the *in vivo* adaptive immune response. First, the collected fresh human blood was processed into T cells and B cells and then co-cultured with dendritic cells to stimulate a lift node that initiates adaptive immune responses. When the dendritic cells present their antigen to the right receptor that matches T cells, the antigen activates T cells, which then activates B cells. These B cells subsequently differentiate into plasma cells, generating antibodies and cytokines.

Figure-1 depicts the conduct of the PTE module. 5 × 10^5^ human umbilical vein endothelial cells were grown to confluency on 24-well plates containing a collagen matrix (PureCol; Advanced Biomatrix, San Diego, CA, USA) using Falcon cell culture inserts (Corning, USA). 5 × 10^5^ monocytes in RPMI medium with 20% human autologous serum and 1% antibiotics (penicillin-streptomycin) were added to each HUVEC well. 24-well plates’ bottom chambers were filled with RPMI medium combined with 20% human autologous serum, 10% DC Generation Medium DXF (PromoCell, Heidelberg, Germany), 1% antibiotic (penicillin-streptomycin) solution, and either 60 μg/mL EBNE or 2.5 μg/mL concanavalin A (Sigma, USA). The co-culture plates were incubated for 90 min at 37°C with 5% CO_2_ in humidified chambers. After 90 min, the non-migrated cells were discarded, and the co-culture was continued for an additional 48 h. 1 μg/mL of lipopolysaccharide (LPS) (Sigma-Aldrich, USA) was added to the co-culture after 48 h and incubated for 24 h. Reverse transmigrated DCs were employed in a co-culture setup to serve as the LTE module (Figure-2) [20–22].

### Transendothelial migration and reverse transendothelial migration cell morphological observations

90 min, 48 h post-treatment with or without EBNE or con A, and 24 h after LPS treatment, monocyte bright-field images were captured using a Nikon inverted microscope (Japan). Monocyte morphology changes were assessed by observing their degree of cellular migration. 1 μg/mL LPS-laden at upper chamber-induced monocytes transformed into DCs upon reverse transmigration through the HUVEC monolayer [20–22].

### Determination of reverse transmigrated and differentiated DCs by flow cytometry

The negative controls, consisting of antibodies labeled with CD14-FITC, CD109-PE, CD86-APC, CD83-APC-vio77, REA Control – FITC, REA Control–PE, REA Control–APC, and REA Control–APC-vio77, were employed to confirm the reverse transmigration and differentiation of DCs. The supernatant was aspirated after centrifuging the cells at 300× *g* for 10 min. The cell pellet was suspended in 98 μL of buffer and then added 2 μL of appropriate antibodies. The cell suspension was incubated in the dark on ice for 10 min. The supernatant was completely aspirated after the cells were centrifuged at 300× *g* for 10 min and washed with 2 mL of buffer. One mL buffer was used to suspend the cell pellet before flow cytometry analysis.

### *In vitro* LTE module preparation

Figure-2 illustrates the implementation of the LTE module. 24-well plates were filled with fifth-passage human umbilical vein endothelial cell monolayers on collagen matrices. The purified T- and B-cells, DCs reverse-transmigrated from human blood, were cultured on a HUVEC monolayer in RPMI medium with 20 % human autologous serum, 1% antibiotics (penicillin-streptomycin), and either EBNE or 2.5 μg/mL of con A. The cell culture was incubated at 37°C, 5% CO_2_, and high humidity for 10 days to simulate the adaptive immune response. After 10 days of co-culture incubation, the supernatants were harvested for immunoglobulin (Ig) and cytokine profiling [20–22].

### Determination of cytokine profiling through cytokine antibody array

RayBiotech’s G-Series 2000 Human Cytokine Antibody Array can detect up to 174 cytokines with high sensitivity using a laser scanner. The study’s four samples underwent the procedure as instructed by RayBiotech, Georgia, USA, for assessing cytokine expression levels. Mean signal intensity values for each cytokine are given, accounting for four standard deviations (SDs).

### Determination of Ig Isotypes Using an Ig Isotype Array

RayBiotech’s Human Ig Isotype Array is a sandwich-enzyme-linked immunosorbent assay (ELISA)-based technology for measuring eight human Ig isotypes (IgG1, IgG2, IgG3, IgG4, IgA, IgD, IgE, and IgM) through fluorescence detection. The evaluation of Ig subclass expression levels from four study samples was carried out following the manufacturer’s guidelines. The mean value and standard deviation for each cytokine’s signal intensity are stated.

### Statistical analysis

Values were averaged using the mean of four standard deviations. One-way analysis of variance with Tukey’s range test was employed to compare average values. 0.05-level p-values indicated statistical significance using VASSARSTATS software (http://vassarstats.net/).

## Results

### Preparation of EBN extracts by aqueous extraction

**Figure-3:**
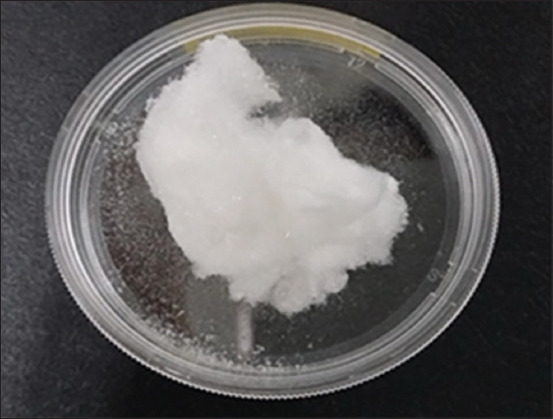
Aqueous extract of edible bird’s nest.

The aqueous extraction method was carried out on the house-cultivated EBN. The resulting extract is illustrated in Figure-3.

### Effect of WST-8 assay on the survival rate of differentiated immune cells

The consequences of varying EBNE concentrations on DCs are tabulated in Table-1. Using the WST-8 assay, the survival rate increased by 42.26% at the optimal concentration of 60 μg/mL of EBNE.

**Table-1:** Effects of different EBNE concentrations on dendritic cell growth.

EBNE concentration (g/mL)	0	20	40	60	80	100
Cell survival rate (%)	100	89.21	109.23	142.26	101.93	96.39

EBNE=Edible bird’s nest aqueous extract

### Phenotypic analysis of monocyte populations

**Figure-4:**
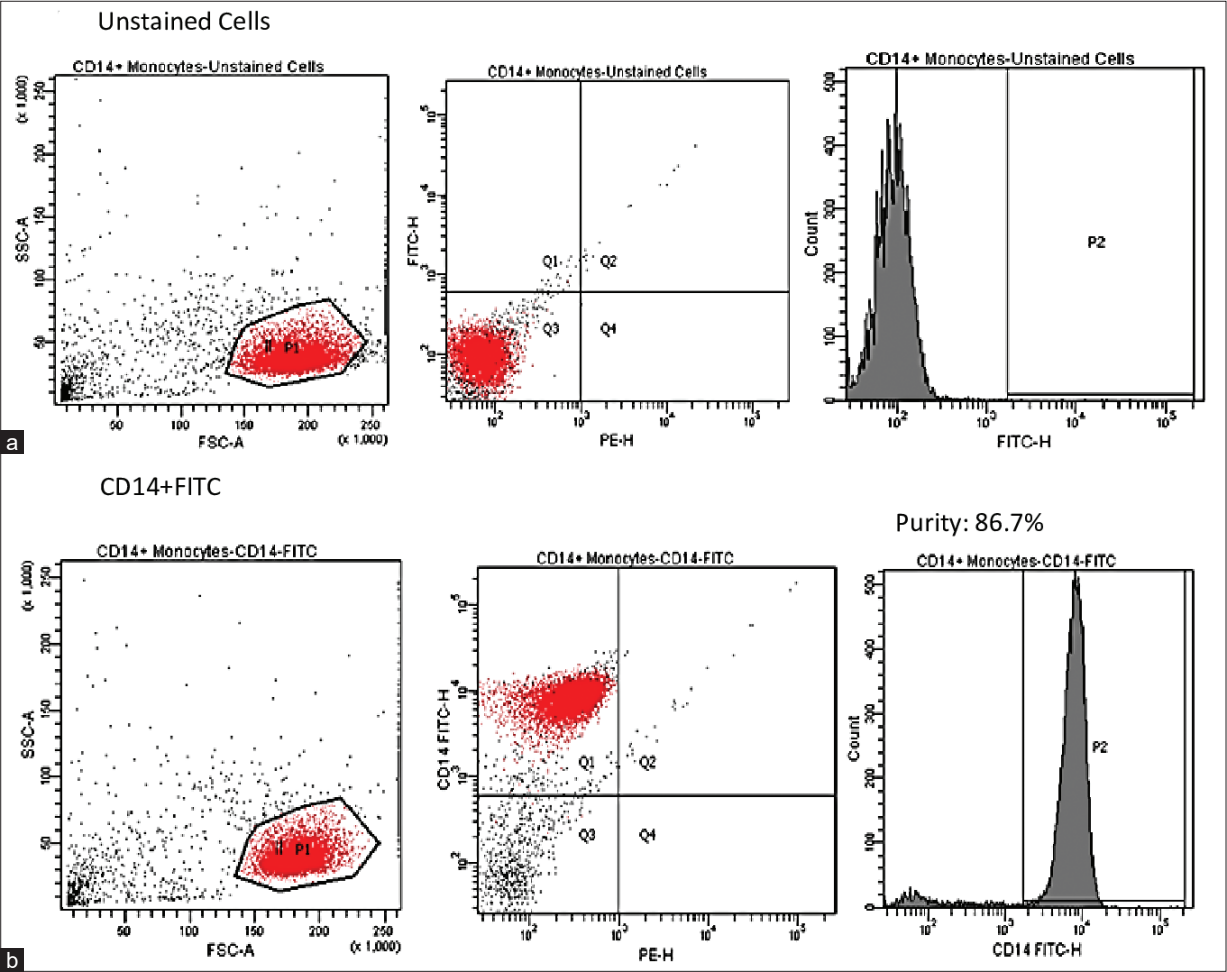
Phenotype and scatter profiles of monocytes after human monocytes preparation through StraightFrom™ Whole Blood CD14 MicroBeads. The expression of CD14^+^ Monocytes was detected by flow cytometry. (a) Unstained cells. (b) Characterization of CD 14^+^ monocytes.

Magnetic-activated cell sorting (MACS)-isolated monocytes were analyzed by flow cytometry for the expression of CD14 surface markers, with the aim of examining the purity of MACS-isolated monocytes. Figure-4 shows that MACS-isolated monocytes express high purity of CD14^+^, which is 86.7% from the histogram.

### Morphological examination of EBNE-treated PTE module cultures

**Figure-5:**
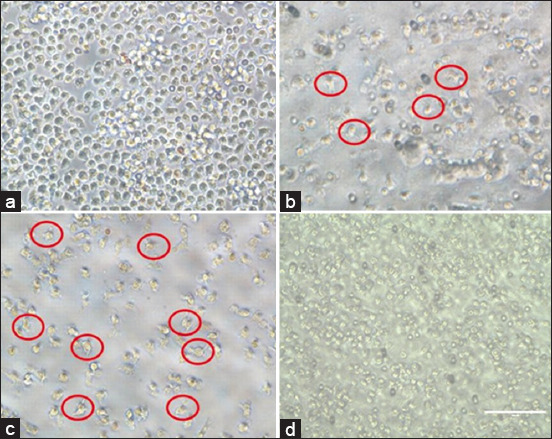
Morphological examination of edible bird’s nest aqueous extract (EBNE)-treated module cultures. (a) Transendothelial migration cells in the peripheral tissue equivalent (PTE) module after 24 h (bottom well), (b) Transendothelial migration cells in the peripheral tissue equivalent (PTE) module after 24 h (upper well), (c) Reverse-transendothelial migration cells in the PTE module after 48 h (bottom well), (d) Reverse-transendothelial migration cells in the PTE module after 48 h (upper well) at the optimal concentration of EBNE, 60 μg/mL. Images taken at a magnification of 40. The red circle denotes the morphology of dendritic-like differentiated cells in terms of hairy-like dendritic cells.

Microscopic examinations reveal transendothelial and reverse transendothelial migration in the PTE module post-EBNE treatment (Figures-5a–d). The morphology of dendritic-like cells, as viewed under the microscope, shows distinct features.

### Phenotypic analysis of the immature and mature DC population

**Figure-6:**
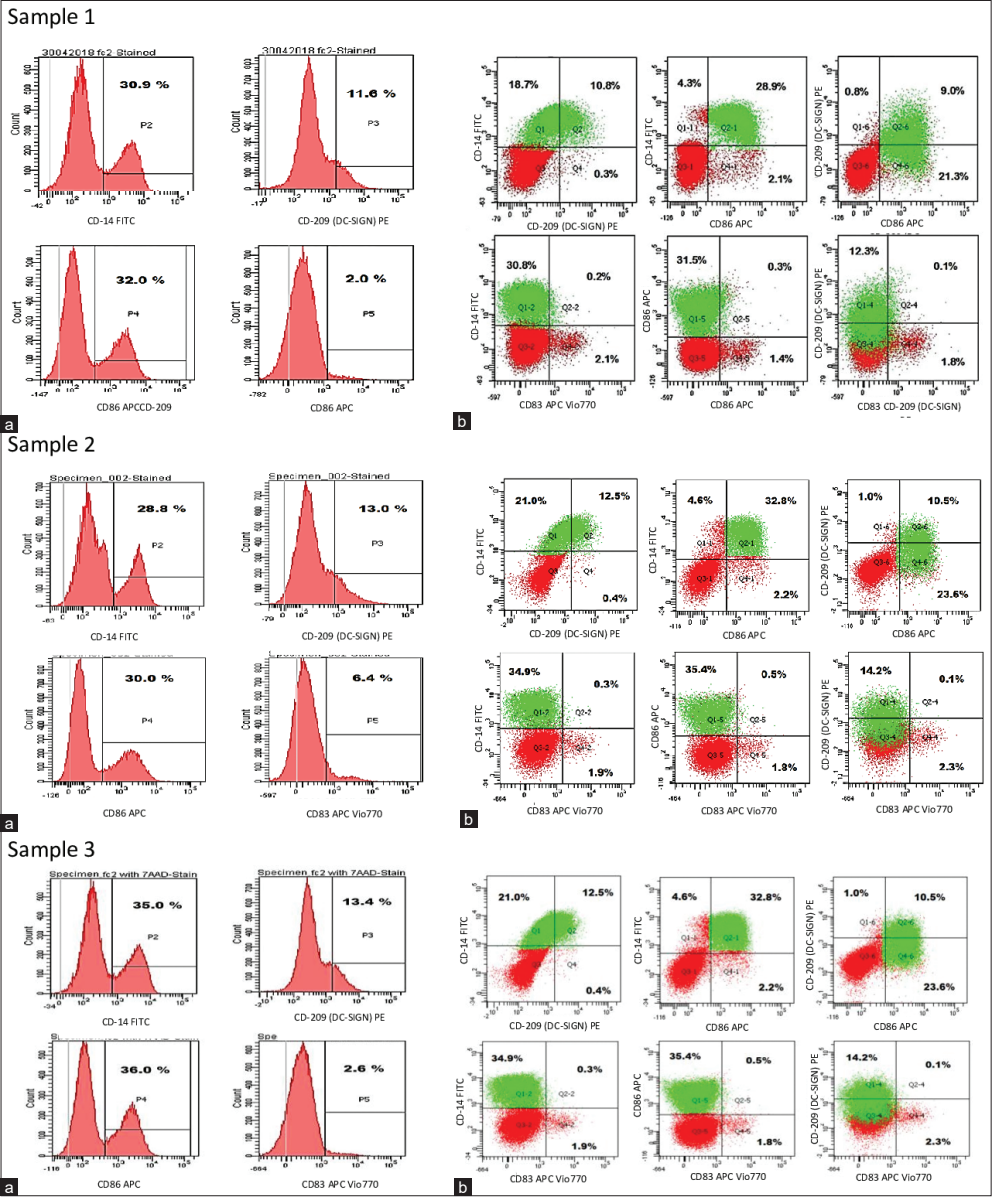
(a) Phenotype and (b) scatter profiles of monocyte-derived immature and mature dendritic cells (DCs) from the peripheral tissue equivalent module (innate immunity). The expression of CD14, CD209 (DC-SIGN), CD86, and CD83 in immature and mature DCs was detected by flow cytometry. Results were obtained from samples 1, 2, and 3.

Figure-6 shows differentiated cells harvested from reverse-transendothelial migration in the PTE module after 48 h, expressing CD14^+^ immature DC, CD109^+^ immature and mature DC, CD86^+^ mature DC, and CD83^+^ mature DC from 3 different individual samples to obtain an average expression of DC markers. The differentiated cells expressed an average of 31.57% of CD14^+^ immature DCs or monocytes, 12.67% of CD209^+^ both immature DCs and mature DCs, 32.67% of CD86^+^ mature DCs, and 3.67% of CD83^+^ mature DCs.

### Phenotypic analysis of the T- and B-cell population

**Figure-7:**
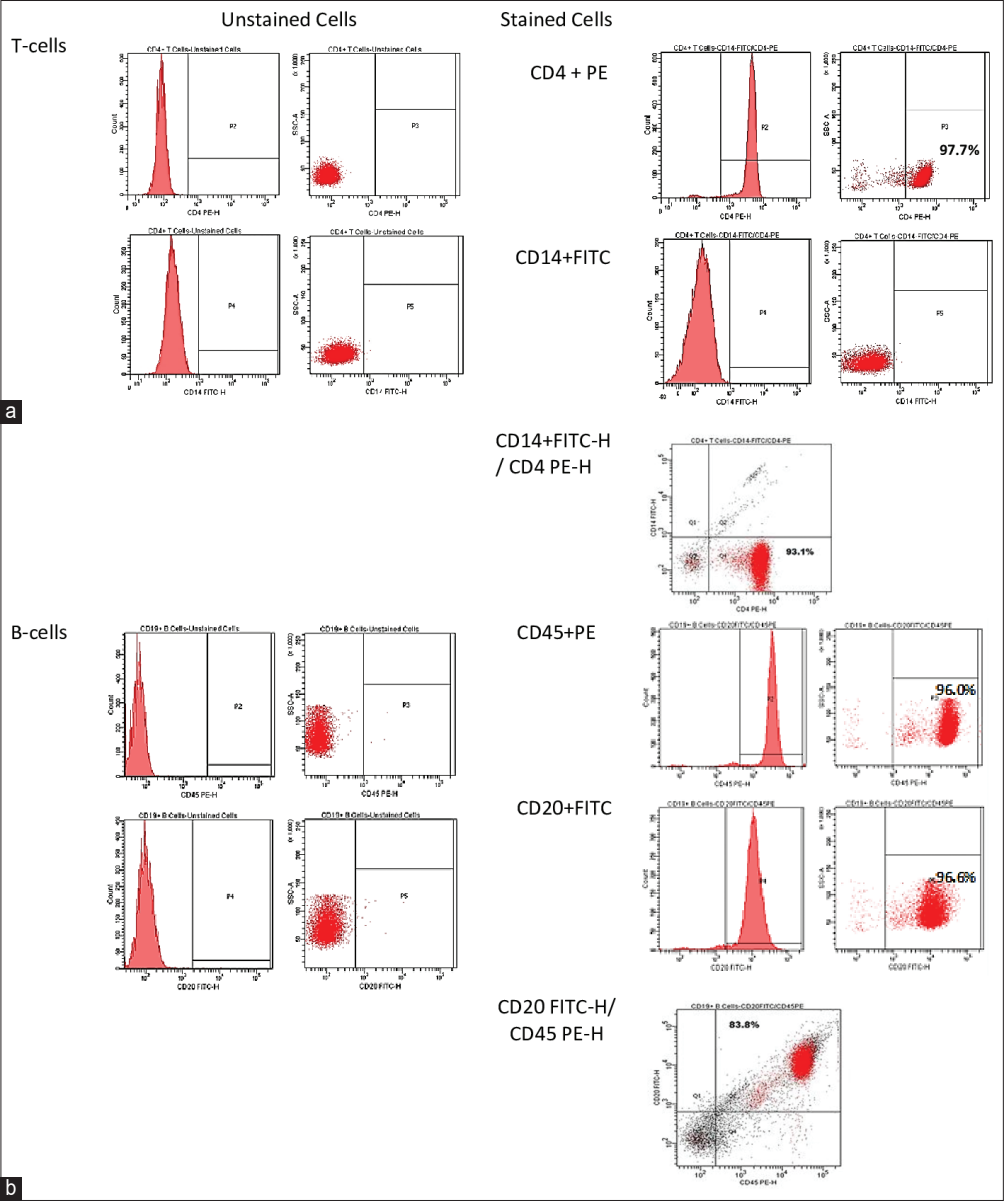
Phenotype and scatter profiles of MACS-isolated T and B cells. (a) Characterization of CD4+ T cells. (b) Characterization of CD20+/CD45+ B cells.

MACS-isolated T- and B-cells were analyzed by flow cytometry for the expression of certain surface markers, with the aim of examining the purity of MACS-isolated T- and B-cells (Figure-7). Figure-7a shows that MACS-isolated T cells express significantly high levels of CD4^+^ (97.7%) and extremely low levels of CD14^+^. The purity of the isolated T cells was 93.1%. MACS-isolated B cells express significantly high levels of CD45^+^ and CD20^+^ at 96.0% and 96.6%, respectively. The purity of the isolated B cells was 83.8%, as shown in Figure-7b.

### EBNE on cytokine expression

**Figure-8:**
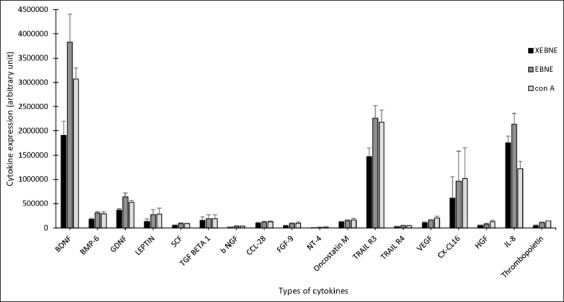
Effects of edible bird’s nest aqueous extract (EBNE) on cytokine expression compared with XEBNE (without EBNE) and concanavalin A (treatment with concanavalin A as control). Values are expressed as Arbitrary Unit Mean ± Standard Deviation of four independent experiments. Brain-derived neurotrophic factor, bone morphogenetic protein 6, glial cell line-derived neurotrophic factor, leptin, stem cell factor, transforming growth factor beta 1, basic nerve growth factor, chemokine (C-C motif) ligand 28, fibroblast growth factor 9, neurotrophin 4, tumor necrosis factor-related apoptosis-inducing ligand R3, tumor necrosis factor-related apoptosis-inducing ligand R4, vascular endothelial growth factor and chemokine (C-C motif) ligand 16, hepatocyte growth factor, and interleukin 8.

**Figure-9:**
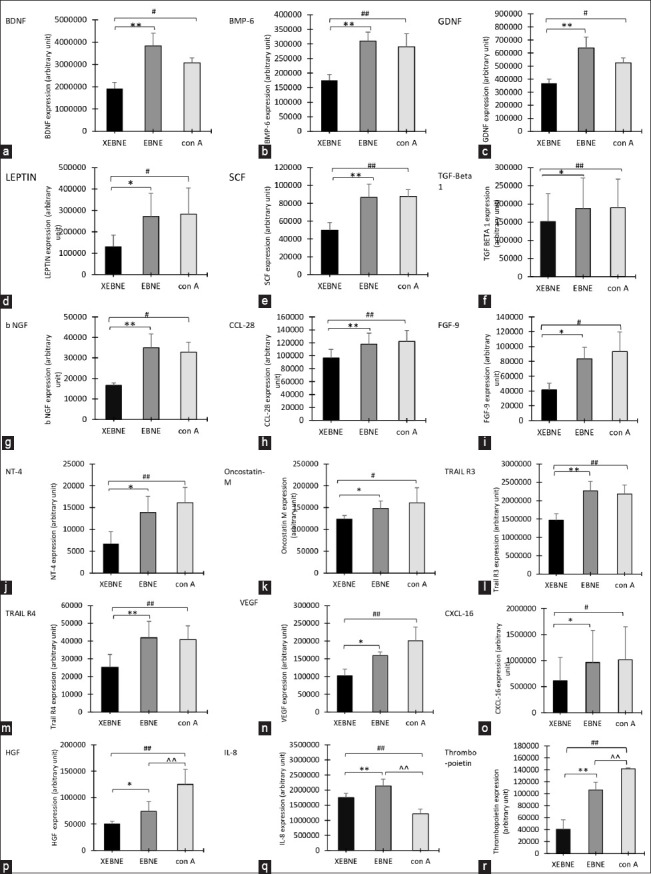
Effects of 60 μg/mL of edible bird’s nest (EBN) extracts on cytokine expression compared with XEBNE (without EBN aqueous extract [EBNE]) and 60 μg/mL of con A (treatment with concanavalin A as control. Cytokine expression of (a) Brain-Derived Neurotrophic Factor (BDNF), (b) Bone Morphogenetic Protein 6 (BMP-6), (c) Glial Cell Line-Derived Neurotrophic Factor (GDNF), (d) Leptin, (e) Stem Cell Factor (SCF), (f) Transforming Growth Factor Beta 1 (TGF- ▪1), (g) Basic Nerve Growth Factor (bNGF), (h) Chemokine (C-C motif) Ligand 28 (CCL28), (i) Fibroblast Growth Factor 9 (FGF9), (j) Neurotrophin 4 (NT-4), (k) Oncostatin M, (l) Tumour Necrosis Factor (TNF)-Related Apoptosis Inducing Ligand R3 (TRAIL R3), (m) Tumour Necrosis Factor (TNF)-Related Apoptosis-Inducing Ligand R4 (TRAIL R4), (n) Vascular Endothelial Growth Factor (VEGF), (o) Chemokine (C-C motif) Ligand 16 (CXCL-16), (p) Hepatocyte Growth Factor (HGF), (q) Interleukin 8 (IL-8) and (r) Thrombopoietin. Values are expressed as Arbitrary Unit Mean ± Standard Deviation of four independent experiments. *Significant difference compared with without EBN extracts (XEBNE) group and with EBN extracts (EBNE) group (p < 0.5). **Significant compared with without EBN extracts (XEBNE) group and with EBN extracts (EBNE) group (p < 0.01). #Significant difference compared with without EBN extracts (non-EBNE) group and with con A (treatment with Concanavalin A as control) group (p < 0.5). ##Significant compared with without EBN extracts (XEBNE) group and with con A (treatment with Concanavalin A as control) group (p < 0.01). ^^Significant compared with with EBN extracts (EBNE) group and with con A (treatment with Concanavalin A as control) group (p < 0.01).

The effects of EBNE on B-lymphocyte cytokine expression are shown in Figure-8. Eighteen cytokines were significantly expressed. Significant increases in brain-derived neurotrophic factor (BDNF), bone morphogenetic protein 6 (BMP-6), glial cell line-derived neurotrophic factor (GDNF), leptin, stem cell factor (SCF), transforming growth factor-beta 1 (TGF-β1), basic nerve growth factor (bNGF), chemokine (C-C motif) ligand 28 (CCL28), fibroblast growth factor 9 (FGF9), neurotrophin 4 (NT-4), oncostatin M, tumor necrosis factor (TNF)-related apoptosis-inducing ligand R3 (TRAIL R3), tumor necrosis factor (TNF)-related apoptosis-inducing ligand R4 (TRAIL R4), vascular endothelial growth factor (VEGF), and chemokine (C-C motif) ligand 16 (CXCL-16) were found between non-EBNE versus EBNE and non-EBNE versus con A, whereas hepatocyte growth factor (HGF), interleukin 8 (IL-8), and thrombopoietin were significant between non-EBNE versus EBNE and non-EBNE versus con A and EBNE versus con A (Figure-9a-r). Table-2 summarizes the functions of the cytokines expressed in this study.

**Table-2:** Categorization of cytokines expressed based on their function.

Function	Cytokines
Pro-neurogenic cytokines	• Brain-derived neurotrophic factor
	• Glial cell line-derived neurotrophic factor
	• Basic Nerve Growth Factor
	• Neurotrophin 4
Tissue and cell promoter cytokines	• Bone morphogenetic protein 6
	• Stem cell factor
	• Fibroblast growth factor 9
	• Hepatocyte growth factor
	• Thrombopoietin
Pro- and anti-inflammatory cytokines	• Transforming growth factor beta 1
	• Leptin
	• Oncostatin M
	• Tumor necrosis factor-related apoptosis-inducing ligand R3
	• Tumor necrosis factor-related apoptosis-inducing ligand R4
	• Vascular endothelial growth factor
	• Chemokine (C-C motif) Ligand 16
Chemo-attractant cytokines	• Chemokine (C-C motif) Ligand 28
	• Interleukin 8

### Effect of EBNE on the production of Ig

**Figure-10:**
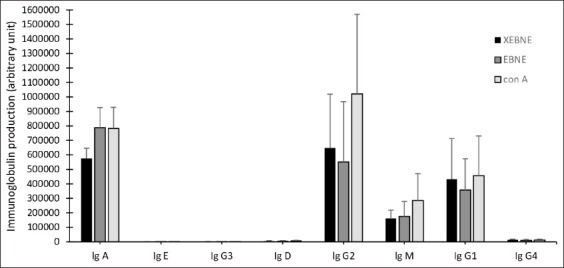
Effects of 60 μg/mL edible bird’s nest (EBN) extracts on the production of immunoglobulin isotypes of B lymphocytes compared with non-EBN aqueous extract (EBNE) (without EBNE) and 2.5 μg/mL concanavalin A (treatment with concanavalin A as control). Values are expressed as Arbitrary Unit Mean ± Standard Deviation of four independent experiments.

**Figure-11:**
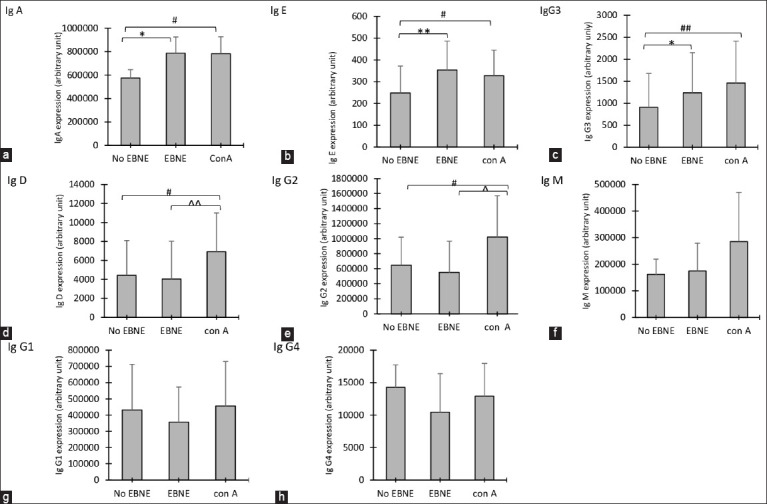
Effects of 60 μg/mL of edible bird’s nest (EBN) extracts on the production of immunoglobulin (a) (Ig)A, (b) Ig-E, (c) IgG3, (d) IgD, (e) IgG2, (f) IgM, (g) lgG1 and (h) IgG4 in comparison with non-EBN aqueous extract (EBNE) (without EBNE) and con A (treatment with Concanavalin A as control. Values are expressed as Arbitrary Unit Mean ± Standard Deviation of four independent experiments. *Significant difference compared with without EBN extracts (XEBNE) group and with EBN extracts (EBNE) group (p < 0.5). **Significant compared with without EBN extracts (XEBNE) group and with EBN extracts (EBNE) group (p < 0.01). #Significant difference compared with without EBN extracts (EBNE) group and with ConA (treatment with Concanavalin A as control) group (p < 0.5). ##Significant compared with without EBN extracts (XEBNE) group and with con A (treatment with Concanavalin A as control) group (p < 0.01). ^Significant compared with EBN extracts (EBNE) group and with con A (treatment with Concanavalin A as control) group (p < 0.5). ^^Significant compared with EBN extracts (EBNE) group and with con A (treatment with Concanavalin A as control) group (p < 0.01).

The effects of EBNE on the Ig isotypes of B-lymphocytes are shown in Figure-10. To investigate the immunomodulatory effects of EBNE, antibody levels were quantified using a sandwich-ELISA-based technology for quantitative measurement of the eight human isotype Igs using a fluorescence-based detection method. The EBNE-treated groups in Figure-11 show increased IgA, IgE, and IgG3 levels significantly between non-EBNE versus EBNE and non-EBNE versus con A, whereas IgD and IgG2 showed a significant increase between non-EBNE versus con A and a significant difference in EBNE and con A. Meanwhile, the changes in the IgM, IgG1, and IgG4 levels are not significant compared with those in the control group.

## Discussion

A model that replicates the human immune system is essential for assessing the impact of EBN on human immunity. This study employed a new biomimetic model of the human immune system to gain comprehensive insights into the EBN-human immune response interactions and mechanisms. The MIMIC model, which includes PTE, LTE, and functional assay modules, is extensively employed as a biomimetic representation of the human immune system [20]. Innate immune responses are primarily observed in the PTE module, with the capacity to MIMIC multiple mucosal surface types as well as different antigen delivery sites. LTE responses recapitulate *in vivo* adaptive immune responses when provided with suitable cells at the appropriate time and under appropriate conditions to permit effective antibody production and/or T-cell responses to vaccines, biologics, or pathogens [21, 22]. Functional assays will confirm the efficacy of these antibodies or T cells against the stimulating antigen. These methods encompass cytokine production, *in vitro* titer enhancement, viral neutralization, and cytotoxic T-cell assays. Hence, these immunity models allow testing for a variety of demographic groupings, such as HLA typing, gender or age biases, and geographic regional differences [20].

TCM considers EBN as an excellent “food tonic” for centuries. The highly esteemed value of EBN is mostly attributed to its high nutritional properties. In general, the nest is shown to contain mostly protein (62–63%), followed by carbohydrates (25.62–27.26%), ash (2.1%), lipids (0.14–1.28%), and minerals [5, 23–25].

The major component of EBN is composed mainly of mucinous glycoproteins (heavily glycosylated proteins) [5], which are rich in sialic acid, N-acetylgalactosamine, N-acetylglucosamine, proteoglycan, glycosaminoglycans, iduronic acid, glucuronic acid, glucose, galactose, fucose, mannose, xylose, rhamnose, hexosamine, hexose, and mannitose [26–31]. EBN also contains all eighteen types of amino acids, including the eight essential amino acids (methionine, threonine, phenylalanine, isoleucine, tryptophan, lysine, valine, and leucine) [5, 24]; essential trace elements such as calcium, sodium, potassium, magnesium, phosphorus, zinc, selenium, manganese, and iron [5, 23, 24]; and EGF [14].

The medicinal benefits of the effect of EBN have been validated based on its multiple biological activities, such as improving bone strength [32, 33], promoting cellular proliferation and tissue regeneration [14, 34–37], anti-inflammation [34, 37, 38], anti-viral effect [6, 39, 40], attenuating oxidative stress [41–43], neuroprotective effect [16, 44–46], and improving sexual dysfunction [47, 48].

The present study investigated how EBN influences human immunity by stimulating monocytes, DCs, T-cells, and B-cells in both the innate and adaptive immune responses. Co-culturing T-cells and B-cells from healthy donors devoid of chronic inflammatory diseases allowed for the examination of EBN’s immunoregulatory effect on the human immune system.

This study found that EBNE enhanced human immunity by boosting the production of specific cytokines and Ig isotypes through faster B-cell multiplication. Applying EBNE can boost both the innate and adaptive immune responses in the equivalent tissue modules (peripheral and lymphoid).

EBNE treatment resulted in the significant expression of eighteen distinct cytokines. The production of various cytokines, including BDNF, bNGF, GDNF, NT-4, SCF, FGF-9, HGF, BMP-6, thrombopoietin, TGF-β1, leptin, oncostatin M, VEGF, CXCL-16, TRAIL R3, TRAIL R4, IL-8, and CCL-28 is raised. DCs and macrophages are the primary producers of the potent signaling cytokines in innate immunity. During the immunological response, cytokines expressed could be redundant (different cytokines having similar functions), pleiotropic (different functions on different target cells), antagonistic (one cytokine executes a particular function while another cytokine inhibits it), synergistic (multiple cytokines strengthen a particular effect), or multifunctional (same cytokines having multiple different functions) [49].

This study’s findings of heightened pro-neurogenic cytokine levels suggest the potential for EBNE to mitigate chronic neuroinflammation common in neurodegenerative diseases. Multiple neurodegenerative diseases, including Parkinson’s and Alzheimer’s, have been linked to diminished BDNF expression [50–52]. Previous studies suggest that BDNF and NT-4 facilitate separate neuronal functions through the activation of distinct receptors like TrkB [53, 54]. GDNF prevented motor neuron degeneration in animal models of amyotrophic lateral sclerosis (ALS), making it a potential biomarker for predicting ALS development [55–57].

The increased expression of the immunomodulatory cytokine VEGF, induced by EBNE-activated nuclear factor κB and activator protein 1, leads to human adipose-derived stem cell proliferation through p44/42 mitogen-activated protein kinase (MAPK) and p38 MAPK pathways. [35]. Rehman *et al*. [58] found hypoxia-induced angiogenic and antiapoptotic factors such as VEGF and HGF secretion from human adipose stromal cells, promoting cell growth and inhibiting apoptosis. In conjunction with previous research [59], this study vividly portrays EBNE’s role in cell proliferation, potentially influenced by VEGF and additional growth and proliferation cytokines, including SCF, HGF, FGF-9, and BMP-6.

IL-8, a chemoattractant cytokine induced by EBNE and known for its ability to activate neutrophils, monocytes, and macrophages, also impedes the adhesion of leukocytes to endothelial cells, demonstrating anti-inflammatory activities [59, 60]. IL-8 promotes both α-smooth muscle actin production in human fibroblasts and acts as a chemotactic agent. During wound healing, fibroblasts enhance migration and collagen deposition through tenascin and fibronectin secretion [61].

Against influenza A virus (IAV), EBN lowered IAV copy number and augmented IL2, IFNγ, NFκB, TNFα, IL6, IL1β, IL4, IL10, IL12, IL27, and CCL2 production. The primary effect of EBN on the body was altering its cytokine patterns, thereby conferring antiviral activity [40]. In disease models other than the current one, EBN reduces oxidative stress resulting in B cell proliferation and increased antibody secretion. In a recent study [39], the antiviral and anti-inflammatory effects of EBN, its chemical constituents from different preparation techniques, and corresponding drugs used for influenza and coronavirus infections were compared. EBN, due to its therapeutic properties, is a promising candidate for investigation in protecting against aerosol transmissible illnesses, particularly during pandemics like coronavirus disease 2019, where treatment drugs are unknown.

We further explored the Ig isotypes to gain a comprehensive understanding of how EBNE influences its mechanism. Igs, acting as antibodies, are mainly found in the blood, tissue fluid, and secretions. A humoral immune response is indicated by these proteins. Igs are classified into IgG, IgM, IgA, IgD, and IgE based on their distinct functions. These findings indicate an increase in IgG, IgG3, IgA, IgE, and IgD levels following EBNE administration. IgM, IgG1, and IgG4 remained unchanged.

Plasma cells in the lymph nodes and spleen are primarily responsible for the synthesis and secretion of IgG. IgG consists of four subclasses – IgG1, IgG2, IgG3, and IgG4. IgG acts as the primary antibody for humoral immunity and carries out substantial homeostatic functions in the body. The production of IgG2 and IgG3 is enhanced by EBNE, while IgG1 and IgG4 remain unaffected. The findings indicate a partial role of EBNE in the humoral immune response. In mucosal immunity, IgA acts as the primary antibody of defense. The study revealed a significant enhancement in IgA secretion due to EBNE treatment. A rise in IgE levels in response to EBNE treatment means more IgE binds to high-affinity receptor for immunoglobulin E (FχεRI) and low-affinity receptor for immunoglobulin E (FχεRII)/CD23, promoting antigen internalization, cell surface presentation, cytokine production, and the elicitation of TH2 responses [62, 63].

This study’s findings of elevated IgD levels could influence humoral immune responses. IgD signals stimulate the activation of B cells, contributing to immune defense against pathogens. IgD’s role is yet to be definitively determined. In mice, EBNE enhanced B-cell proliferation and activation, resulting in increased levels of IgM, IgA, and IgG3 and reduced intestinal immune injury [64]. A previous study by Zhang *et al*. [15] reported an increase in IgM due to increased T-lymphocyte transformation in mice given pearl EBN extract. Dai *et al*. [65] found that EBN augmented T-lymphocyte transformation and IgM levels in mice. Our study showed no significant impact of EBN extract on IgM levels. The findings from this study collectively suggest that EBNE enhances human immune function.

## Conclusion

For the first time, this study has shown that upregulating cytokines, such as BDNF, bNGF, GDNF, NT-4, SCF, IL-4, IL-6, TGF-β1, leptin, FGF-9, oncostatin M, TRAIL R3, TRAIL R4, VEGF, CXCL-16, HGF, IL-8, and CCL-28, as well as Ig levels of IgG, IgG3, IgA, IgE, and IgD, significantly enhance human immunity. The immunomodulatory effects of EBNE could be beneficial for immune-suppressed diseases as an adjuvant therapy. The results of this study necessitate further mechanistic investigations to validate them.

## Authors’ Contributions

YML: Conceptualization, data analysis and interpretation, manuscript drafting, editing, revision, and supervised the study. MJC: Sample collection, laboratory test, and manuscript drafting, editing, and revision. HDD, LCL, SDS, CHT, and SKC: Conceptualization, data analysis and interpretation, and manuscript drafting, editing, and revision.All authors have read, reviewed, and approved the final version of the manuscript.
